# Rare Noncoding Mutations Extend the Mutational Spectrum in the *PGAP3* Subtype of Hyperphosphatasia with Mental Retardation Syndrome

**DOI:** 10.1002/humu.23006

**Published:** 2016-05-19

**Authors:** Alexej Knaus, Tomonari Awaya, Ingo Helbig, Zaid Afawi, Manuela Pendziwiat, Jubran Abu‐Rachma, Miles D. Thompson, David E. Cole, Steve Skinner, Fran Annese, Natalie Canham, Michal R. Schweiger, Peter N. Robinson, Stefan Mundlos, Taroh Kinoshita, Arnold Munnich, Yoshiko Murakami, Denise Horn, Peter M. Krawitz

**Affiliations:** ^1^ Institute for Medical and Human GeneticsCharité‐Universitätsmedizin BerlinAugustenburger Platz 1Berlin13353Germany; ^2^ Max Planck Institute for Molecular GeneticsIhnestr. 63–73Berlin14195Germany; ^3^ Berlin‐Brandenburg School for Regenerative Therapies (BSRT)Charité Campus Virchow KlinikumAugustenburger Platz 1Berlin13353Germany; ^4^ Department of PediatricsKyoto University Graduate School of MedicineSakyoKyoto6068507Japan; ^5^ Division of Child NeurologyThe Children's Hospital of PhiladelphiaPhiladelphiaPennsylvania19104‐4399; ^6^ Department of NeuropediatricsUniversity Medical Center Schleswig‐HolsteinKiel 24105Germany; ^7^ Sackler Faculty of MedicineTel Aviv UniversityTel AvivIsrael; ^8^ Department of Laboratory Medicine and PathobiologyUniversity of TorontoTorontoOntarioCanada; ^9^ Greenwood Genetic CenterGreenwoodSouth Carolina; ^10^ North West Thames Regional Genetics ServiceNorthwick Park HospitalHarrowHA1 3UJUK; ^11^ Epigenomics and Tumor GeneticsCCGUniversity of CologneCologne50931Germany; ^12^ Department of ImmunoregulationResearch Institute for Microbial DiseasesOsaka UniversityOsaka565Japan; ^13^ World Premier International Immunology Frontier Research CenterOsaka UniversityOsaka565Japan; ^14^ Hôpital Necker – Enfants MaladesUnité INSERM 781Laboratoire de génétique moléculaire Tour Lavoisier – 2ème étageParis Cedex15 75743France

**Keywords:** intellectual disability, hyperphosphatasia with mental retardation, Mabry syndrome, noncoding mutations, *PGAP3*

## Abstract

HPMRS or Mabry syndrome is a heterogeneous glycosylphosphatidylinositol (GPI) anchor deficiency that is caused by an impairment of synthesis or maturation of the GPI‐anchor. The expressivity of the clinical features in HPMRS varies from severe syndromic forms with multiple organ malformations to mild nonsyndromic intellectual disability. In about half of the patients with the clinical diagnosis of HPMRS, pathogenic mutations can be identified in the coding region in one of the six genes, one among them is *PGAP3*. In this work, we describe a screening approach with sequence specific baits for transcripts of genes of the GPI pathway that allows the detection of functionally relevant mutations also including introns and the 5′ and 3′ UTR. By this means, we also identified pathogenic noncoding mutations, which increases the diagnostic yield for HPMRS on the basis of intellectual disability and elevated serum alkaline phosphatase. In eight affected individuals from different ethnicities, we found seven novel pathogenic mutations in *PGAP3*. Besides five missense mutations, we identified an intronic mutation, c.558‐10G>A, that causes an aberrant splice product and a mutation in the 3′UTR, c.*559C>T, that is associated with substantially lower mRNA levels. We show that our novel screening approach is a useful rapid detection tool for alterations in genes coding for key components of the GPI pathway.

## INTRODUCTION

Glycosyl phosphatidylinositol (GPI) anchor deficiencies are a new class of congenital disorders of glycosylation that present with a broad phenotypic spectrum that is incompletely understood. The GPI is a glycolipid that anchors more than 150 proteins to the cell surface. At least 27 genes are involved in the biosynthesis and transport of GPI anchored proteins and disease‐causing mutations have been described for 13 of these genes [Kinoshita et al., 2008; Freeze et al., 2014]. Among the most common features in GPI‐anchor deficiencies are intellectual disability, and epilepsies. For Mabry syndrome, a well‐characterized subtype of GPI‐anchor deficiencies, an elevated serum activity of the alkaline phosphatase is an additional characteristic diagnostic marker that initially prompted Mabry and collaborators to postulate a separate syndrome that is now referred to as Hyperphosphatasia with Mental Retardation Syndrome [Mabry et al., 1970; Horn et al., 2010; Thompson et al., 2010]. As of 2016, six subtypes of HPMRS are known (OMIM Phenotypic Series, PS239300) and pathogenic mutations in the genes of the GPI anchor synthesis, *PIGV*, *PIGW, PIGY, PIGO*, as well as of the anchor maturation, *PGAP2* (MIM #614207) and *PGAP3* (MIM #615716), have been identified [Krawitz et al., 2010; Krawitz et al., 2012; Hansen et al., 2013; Krawitz et al., 2013; Chiyonobu et al., 2014; Howard et al., 2014; Ilkovski et al., 2015]. For GPI biosynthesis genes, most of the reported pathogenic variants are missense mutations, but loss of function mutations have also been identified. However, biallelic nonsense or frameshift mutations have not been observed in humans so far, but have only been found in compound heterozygous combinations with missense mutations. It is therefore assumed that all inherited forms of GPI‐anchor deficiencies, IGDs, have a residual activity of GPI‐anchored proteins. This assumption is supported by studies of animal models, as mice incapable of forming GPI‐anchors are not viable [Tarutani et al., 1997; Nozaki et al., 1999].

**Figure 1:**
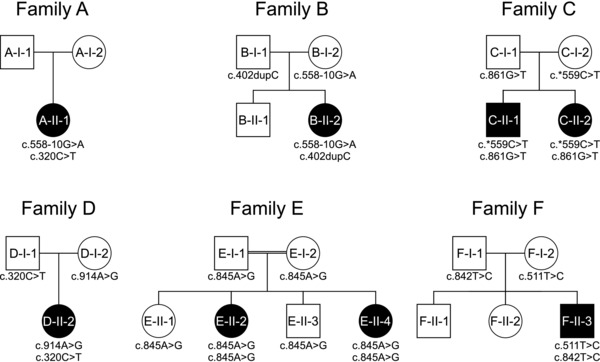
Pedigrees and segregation of identified mutations. Families A, B, C, and D are of European descent, family E is Palestinian, and patient F is from Japan. For detailed clinical information and high‐resolution photographs of patients, see Supporting Information (Supp. Fig. 1–5).

Besides mutations that affect the protein function directly, hypomorphic mutations in the promoters for *PIGM* as well as *PIGY* have recently been reported [Almeida et al., 2006; Ilkovski et al., 2015]. We therefore hypothesize that some of the unsolved cases with Mabry syndrome might also be due to a substantial reduction in the expression of wild‐type transcripts of the known genes. As a result, we refined our GPI‐gene panel screening approach to detect affected individuals on the RNA rather than DNA level. In the current manuscript, we present eight previously unreported patients (Fig. 1) with Mabry syndrome carrying novel mutations in *PGAP3*, a gene of the GPI‐anchor maturation. The expressivity of the phenotype varies considerably for this cohort (Supp. Figs. S1–S5).

## Material and Methods

### Mutation Analysis

Genomic DNA (gDNA) was isolated from fibroblasts or blood of all participants of this study. The Charité University Medicine ethics board approved this study, and we obtained informed consent from the responsible persons (parents) on behalf of all study participants. The DNA was fragmented and a library for high‐throughput sequencing was prepared according to standard procedures. RNA was obtained from fibroblast cultures, fragmented and reversely transcribed into a cDNA library. gDNA and cDNA libraries were enriched with exon‐specific oligonucleotide baits as previously described [Krawitz et al., 2013] and high‐throughput sequencing was performed on the MiSeq platform. All sequence reads were mapped to the genomic reference GRCh37 and processed with a splice junction mapper in case of cDNA. Most reads of the correctly spliced mRNAs were exon‐spanning and fragments mapping to intronic sequences were rare. The coverage of coding exons of all known GPI‐synthesis‐related genes was above 20‐fold in all analyzed samples. Sequence variants in gDNA alignments were detected according to GATK's best practice guidelines. All variant calls from gDNA that are discussed in this manuscript were validated by ABI Sanger sequencing. Differences in transcript‐specific expression were confirmed by quantitative PCR on RNA isolated from fibroblast cultures. qPCR measurements were performed in triplicates from two biological duplicates. dCt values were normalized against GAPDH. Primer pair sequences and qPCR settings are available upon request. All sequence variants described in the manuscript have also been submitted to ClinVar (http://www.ncbi.nlm.nih.gov/clinvar/) and GeneTalk (http://gene‐talk.de/targetlections/35134).

### Characterization of Noncoding Mutations

Short‐read fragments from high‐throughput sequencing were first mapped to the human reference genome GRCh37 with bwa‐mem, and cDNA alignments were further refined with splice junction mapper TopHat. More than 95% of the coding positions of all known genes of the GPI‐anchor synthesis pathway were covered by more than 100 independent sequences. The allele‐specific ratios for RNA‐seq experiments were assessed by considering only high‐quality reads (phred>30, less than three mismatches).

### Cell Culture and Flow Cytometry

Fibroblasts were obtained from deep skin biopsies, treated with collagenase and grown in a Dulbecco's modified Eagle's medium supplemented with 10% fetal bovine serum (FBS), 1% glutamine, 1% penicillin/streptomycin, and grown in a 5% CO_2_ incubator.

For investigation of GPI‐anchored proteins, cultured skin fibroblasts derived from patient A‐II‐1, patient B‐II‐2, patient C‐II‐2, and controls were removed from the dish with cold EDTA and a cell scraper, pipetted several times to get a single cell suspension, counted, washed, and stained with FLAER (fluorescent‐labeled aerolysin [AF‐488]) and antibodies against GPI‐linked proteins (CD55‐PE, CD59‐PE, CD87‐APC, CD73‐PE‐Cy7) in phosphate‐buffered saline without calcium or magnesium and supplemented with 2% FBS for 1 hr at room temperature. Cells were then centrifuged at 300*g* for 3 min and washed twice in the above solution. Labeled cells were analyzed using a CantoII flow cytometer (BD Biosciences San Jose, CA, USA) and FlowJo™ v. 9.8.2 software.

## Results

In all individuals with the minimal diagnostic criteria developmental delay, intellectual disability, and elevated serum alkaline phosphatase, we first analyzed the gDNA of GPI‐anchor pathway genes for coding mutations as previously described [Howard et al., 2014]. At the time of writing, we have 37 individuals who meet these criteria in our ongoing study and were genetically confirmed. In this work, we focus on the molecular findings in patients from six unrelated families, who showed mutations in *PGAP3* (Fig. 1). In three of these families, the results from gDNA sequencing were inconclusive as only one heterozygous coding mutation was identified that qualified as pathogenic, based on current classifications standards. By cDNA sequencing, we could confirm a functional impact for additional noncoding mutations in these patients and we argue that this extended work‐up increases the diagnostic yield. Considering all molecularly confirmed cases of Mabry syndrome in our laboratory over the last 5 years, *PGAP3* has now become the second most prevalent disease gene for this phenotypic series after *PIGV*.

All patients with genetic alterations in *PGAP3* have a combination of severe global developmental delay and elevated levels of alkaline phosphatase activity, which are the most indicative features for HMPRS (Table 1). However, we also observe that there is some variability within the phenotypes and we will therefore describe the clinical features of the patients of this report in detail. Two individuals started to walk at a normal age, but the others did not walk until the age of between 3 and 7 years, and one individual was not able to walk at all at the age of 16 years. Five individuals did not achieve verbal communication, and three other patients used only single words. Furthermore, six individuals developed seizures with variable onset and severity. Muscular hypotonia was found in four individuals. The presence of ataxia or unsteady gait was striking in seven patients (patients of family B, C, D, E, and F).

**Table 1:** Summary of Clinical Findings in Patients Carrying *PGAP3* Mutations

Patient	A‐II‐1	B‐II‐2	C‐II‐1	C‐II‐2	D‐II‐1	E‐II‐2	E‐II‐4	F‐II‐3
Ethnicity	European American	German	French	French	British	Palestinian	Palestinian	Japanese
Consanguinity	No	No	No	No	No	Yes	Yes	No
Age of last assessment (years)	25 years	3 years	5 years	3.5 years	23 years	16 years	10 years	3 years
OFC at birth	35 cm	Normal	+2 SD	+2 SD	Normal	Normal	Normal	Normal
OFC	+3.2 SD	+0.4 SD	+3 SD	+2 SD	+1.2 SD	+0.4 SD	−0.4 SD	Normal
Height	−0.6 SD	+0.4 SD	Normal	Normal	Normal	+0.4 SD	+ 0.4 SD	−2 SD
Weight	BMI 33.3	+2.4 SD	Normal	Normal	Normal	−2.4 SD	−1.4 SD	−2 SD
Global developmental delay	+	+	+	+	+	+	+	+
Motor delay	+	+	+	+	+	+	+	+
Speech and language development	No word	Single words	No word	No word	No word	Single words	Single words	No word
Muscular hypotonia	−	+	−	−	−	+	+	+
Seizures	+	+	+	−	+	+	+	−
Age of onset of seizures (years)	21 years	18 months	24 months	NA	23 years	3½ years	5 years	NA
Type of seizures	Generalized, myoclonic	Myoclonic	Myoclonic	Myoclonic	NA	Not specified	Generalized, myoclonic	NA
AEDs	Lamotrigine	Valproate	Valproate, diazepam	NA	Levetiracetam	Lamotrigine, clonazepam	Clonazepam	NA
Behavioral abnormalities	Sleep disturbance, autism, aggression, OCD	Sleep disturbance	−	−	Autism, mood disorder, hypersomnolence	Sleep disturbance	Sleep disturbance	Sleep disturbance
Other neurological abnormalitites	−	Ataxia	Ataxia, oculomotor apraxia	Ataxia	Unsteady gait	Ataxia, sensorineural hearing loss	Ataxia, sensorineural hearing loss	Ataxia, sensorineural hearing loss
Facial gestalt								
Apparent hypertelorism	−	+	−	−	−	−	−	−
Upslanting palpebral fissures	+	−	−	−	−	−	−	−
Broad nasal bridge	+	+	−	−	+	−	−	−
Broad nasal tip	−	+	−	−	+	+	+	−
Short nose	−	+	−	−	−	−	−	−
Tented upper lip vermilion	+	+	+	+	+	+	+	−
Large, fleshy ear lobes	−	−	−	−	+	−	−	+
Cleft palate	+	−	−	−	+			+
Brachytelephalangy	−	Broad finger and toenails	−	−	Broad second toe nail	Broad finger and toenails	Broad finger and toenails	−
Serum total ALP (U/l)	469	333	830–1,000	830–900	300–738	807	633	1,848–5,275
Upper reference limit in ALP test (U/l)	136	297	410	410	105	297	297	335
Further anomalies	−	Small teeth	−	−	Constipation, intestinal malrotation	−	−	Constipation, severe food allergy, eczema
PGAP3 variants (NM_033419.3)	c.320C>T, c.558‐10G>A	c.402dupC, c.558‐10G>A	c.861G>T, c.*559C>T	c.861G>T, c.*559C>T	c.320C>T, c.914A>G	c.845A>G (hom)	c.845A>G (hom)	c.511T>C, c.842T>C

OFC, occipitofrontal head circumference; SD, standard deviations; BMI, body mass index; ND, no data available; NA, not applicable; OCD, obsessive compulsive disorder; ALP, alkaline phosphatase.

Six patients showed particular behavioral problems. Among them, sleep disturbance was described as most frequently reported feature (patients A, B, E, F); one patient (D) exhibited long‐lasting episodes of hypersomnolence classified as Klein‐Levine syndrome. Additionally, hyperactivity, autistic features, and obsessive‐compulsive disorder were documented in patients A and D. In patient B, MRI revealed a mild brain anomaly, namely, a small capsula interna. The siblings of family E also showed brain atrophy of the temporal lobes. A megacisterna magna was found in patient F. Hearing loss was documented in three individuals (patients from family E and F).

At birth, normal growth parameters and head circumferences were observed in all patients. During infancy and childhood, growth parameters were found to be variable but within the normal range in most patients. However, two individuals showed an increased and one a decreased weight at the last physical examination. The postnatal occipitofrontal head circumference varied between −0.4 and +3.2 SD with three affected individuals who showed macrocephaly. No distinct pattern of facial anomalies was present in this cohort. But five of them showed some facial dysmorphisms compatible with other forms of HPMRS, such as short nose with a broad nasal bridge and tip, as well as a thin and tented upper lip. Apart from broad fingers and toenails in four individuals (families B, D, E), no other anomalies of the hands and feet were found. A cleft palate was documented in three patients (families A, D, F).

Despite a substantial clinical variability in our patient cohort, all patients showed global developmental delay as well as hyperphosphatasia and thus fulfilled the criteria for Mabry syndrome.

**Figure 2:**
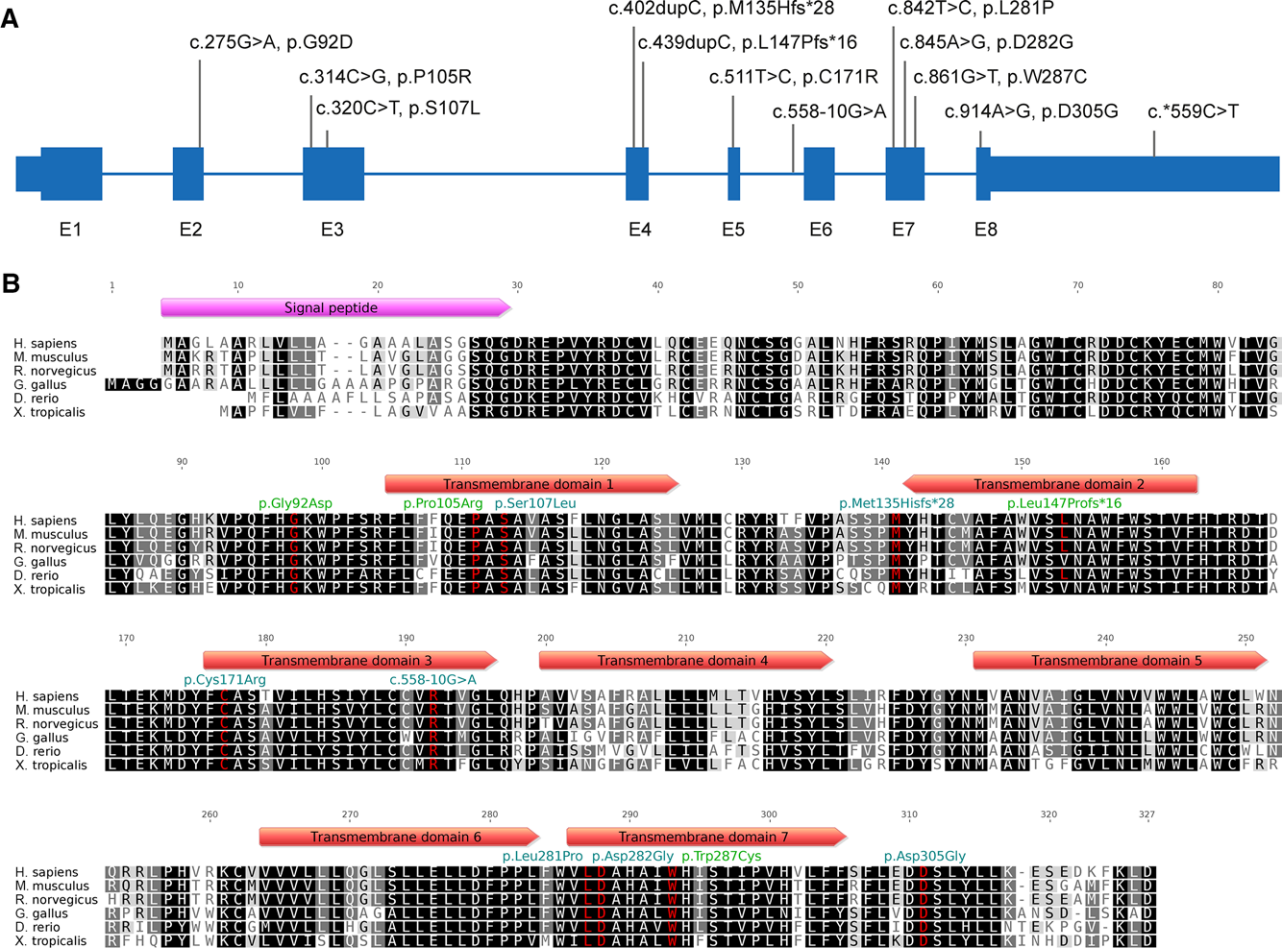
Overview of pathogenic mutations in PGAP3. **A**: The coding sequence of PGAP3 is organized in eight exons. The majority of pathogenic mutations in PGAP3 are missense mutations without a specific pattern for the distribution of pathogenic variants. The intronic mutation c.558‐10G>A impairs the splicing of intron 5 and the 3′UTR mutation c.*559C>T is associated with a reduced expression. Mutation numbering is based on cDNA level. **B**: The protein has seven predicted transmembrane domains (TDs), and the mutations affect highly conserved amino acid residues in TDs as well as in the intracellular and extracellular sites. Green: known pathogenic mutations, cyan: new pathogenic mutations.

All affected individuals were first analyzed for rare coding variants in known exons of the GPI‐anchor synthesis pathway on gDNA as previously described [Krawitz et al., 2013] or through related sequencing techniques. Genomic sequencing alone was able to identify the molecular cause of the disease for the affected individuals in family D, E, and F, identifying the following homozygous or compound heterozygous missense mutations: D‐II‐1: p.Ser107Leu, p.Asp305Gly, E‐II‐3 and E‐II‐4: p.Asp282Gly, F‐II‐1: p.Cys171Arg, p.Trp287Cys (Fig. 2). All these missense mutations caused the decreased activity of PGAP3 (Supp. Fig. S6). This finding also confirms that biallelic missense mutations represent the most common cause of Mabry syndrome in all known disease genes, *PIGV*, *PIGO*, *PGAP2*, and *PGAP3*.

**Figure 3:**
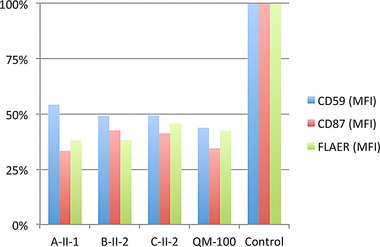
Cell surface levels of GPI markers on fibroblasts. The mean fluorescent intensity (MFI) of the GPI‐anchored proteins CD55 and CD59, as well as FLAER is significantly reduced in fibroblasts of all PGAP3 deficient individuals, compared with pooled healthy controls, confirming a GPI‐anchor deficiency on a functional level. On average, the reduction of marker expression in individuals A‐II‐1, B‐II‐2, and C‐II‐2 is comparable with the reduction of QM‐100 (compound heterozygous carrier of two coding mutations: c.440_441insC, c.914A>G).

In 4/8 patients, only single heterozygous mutations could be identified, that have a detrimental effect on the protein level or its activity (A‐II‐1: p.Ser107Leu, B‐II‐2: p.Met135Hisfs*28, C‐II‐1/2: p.Trp287Cys) (Supp. Fig. S7). As hypomorphic promoter mutations have been described in two other genes of the GPI‐anchor synthesis pathway, *PIGM* and *PIGY*, we hypothesized that the missing second mutations could also be noncoding variants [Almeida et al., 2006; Ilkovski et al., 2015]. In contrast to the classification of coding mutations, predicting the effect of variants that are deeply intronic or in untranslated regions (UTR) is challenging from a bioinformatics perspective. We have therefore further developed our gene‐panel screening approach in such a way that it is suitable for the analysis of transcripts involved in the GPI‐anchor synthesis and remodeling. Before we subjected the samples to a quantitative analysis of cDNA, we confirmed a reduction of GPI‐linker markers on the cell surface (Fig. 3; Supp. Fig. S6).

In the subsequent analysis of the cDNA, we achieved optimal results in target‐specific enrichment with a mean fragment size around 200 bp. Most reads of the correctly spliced mRNAs were exon‐spanning and fragments mapping to intronic sequences were rare.

**Figure 4:**
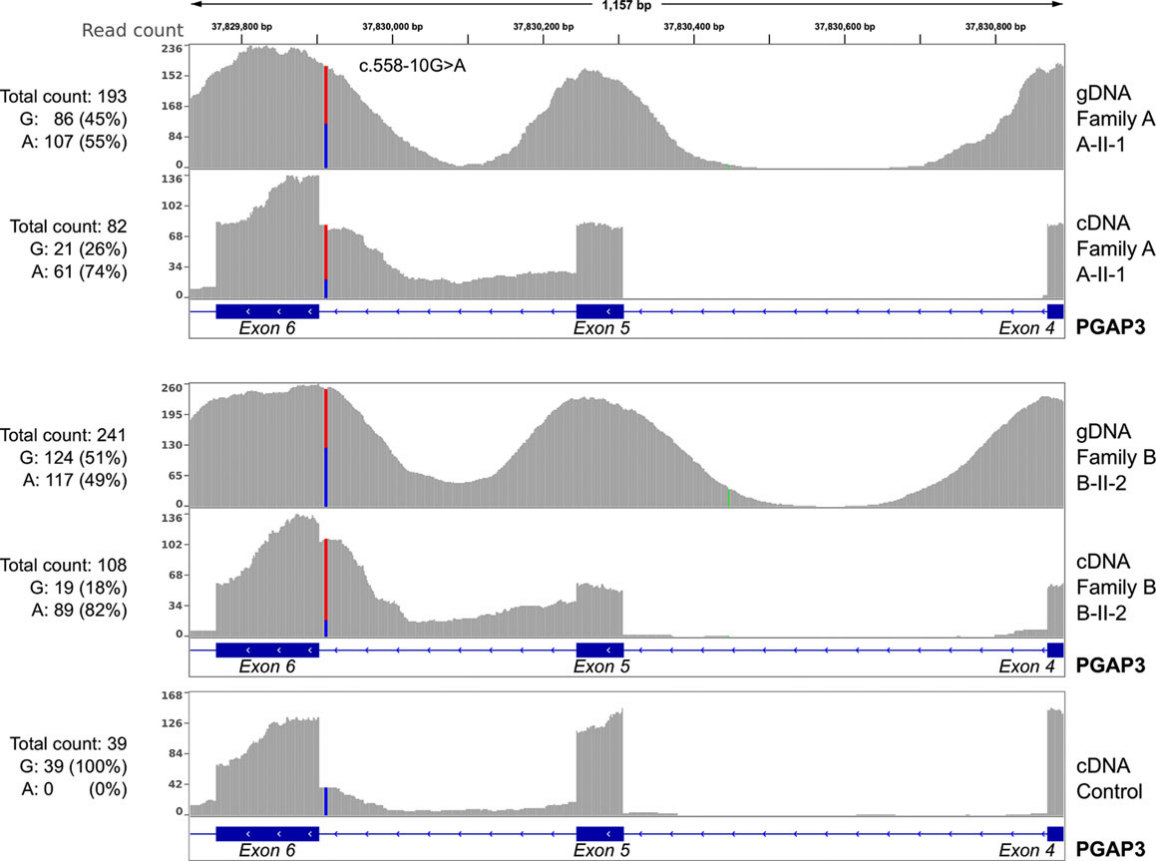
Intronic mutation causing aberrant splicing. The coverage is shown for gDNA and cDNA of PGAP3 for exons 5, 6, and partly 4, as well as for introns 4 and 5 of individuals A‐II‐1 (panel 1 gDNA and panel 2 cDNA), B‐II‐2 (panel 3 gDNA and panel 4 cDNA), and one control (panel 5 cDNA). gDNA sequence reads of the affected individuals of family A and B show the heterozygous intronic mutation c.558‐10G>A with the expected ratio of about 50%. Intron 4 is correctly spliced out in the cDNA of all samples. A low rate of reads map into intron 5 in the healthy control (panel 5), suggesting that intron 5 is not removed in all transcripts. However, the two affected individuals show a high increase of reads from intron 5 in the cDNA. While about half of the enriched fragments of genomic DNA show the c.558‐10G>A mutation, the fraction of the mutation increases to about four‐fifth in the cDNA. The heterozygous mutation c.558‐10G>A thus impedes correct splicing of intron 5 from the pre‐mRNA and results in an aberrant splice product.

A considerable amount of sequence reads that map to intron 5 in *PGAP3* were found in the affected individuals of families A and B. In A‐II‐1, there is a sharp drop in coverage at the intron–exon boundary to almost 50% and in B‐II‐2 to about 70%, indicating that a heterozygous allele is causing a splicing defect. Most of the sequence fragments that reach into intron 5 show the intronic variant c.588‐10G>A, whereas this variant is a clear heterozygous call on the genomic level (Fig. 4). In all heterozygous carriers of the c.588‐10G>A mutation as well as in all healthy controls, there is a low rate of intron 5 fragments with the wild‐type allele c.588‐10G. We therefore assume that even in the wild type, intron 5 is not always spliced out, but that the mutation c.588‐10G>A reduces the splicing efficiency dramatically. The single base pair duplication c.402dupC in the father of the family B (B‐I‐1) results in a premature stop codon. The resulting transcript including the c.402dupC is thus subject to nonsense‐mediated decay, which is confirmed by qPCR expression analysis of PGAP3 in fibroblasts. The second heterozygous mutation of the index (B‐II‐2) c.588‐10G>A results in an expression level of only 10% (Supp. Fig. S8). This also explains the less prominent reduction of coverage in B‐II‐2 at the exon–intron boundary (Fig. 4; panel 4).

**Figure 5:**
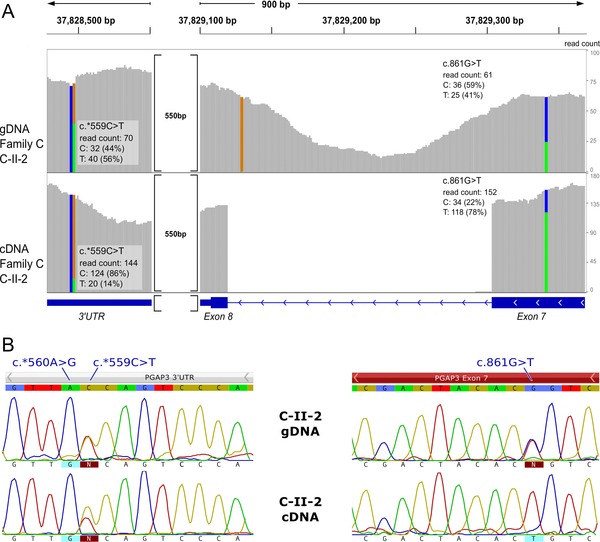
Effect of 3′UTR mutation on transcript level. **A**: The ratio of reads on gDNA of the compound heterozygous mutations c.861G>T and c.*559C>T are comparable (upper panel) in the affected individual from family C. For cDNA data, the allelic balance deviates in opposite directions. On cDNA level, the ratio of the mutant allele at the 3′UTR position drops below one‐fifth, whereas most of the sequence reads show the mutant allele at the position of the missense mutation. **B**: Sanger sequencing of gDNA and cDNA of individual C‐II‐2. A quantitative measurement of transcripts carrying the mutations is not possible via Sanger sequencing.

In affected individuals C‐II‐1 and C‐II‐2, we identified a heterozygous missense mutation in exon 7 of *PGAP3*, c.861G>T, p.Trp287Cys. The only sequence variant that would qualify as pathogenic second *PGAP3* mutation based on its allele frequency is a novel 3′UTR mutation, c.*559C>T. On the cDNA level, this mutation only occurs in less than a fifth of the reads, indicating a more than threefold reduction of this transcript (Fig. 5A). This is in agreement with the contrary ratio of the compound missense mutation c.861G>T that is present in the majority of the sequence reads. The low expression of *PGAP3* for the haplotype with the 3′UTR mutation can be quantified by qPCR from fibroblast RNA. The father, C‐I‐1, who is a heterozygous carrier of the missense mutation p.Trp287Cys, shows expression levels comparable to a healthy control, whereas the index (C‐II‐2) and her mother (C‐I‐2), who are carrier of c.*559C>T, have a markedly reduced expression of *PGAP3* (Supp. Fig. S8).

In summary, we have four patients with noncoding variants in our cohort that are likely to retain a residual level of functional PGAP3. However, we suspected that the amount of wild‐type PGAP3 is pathologically low and in consequence it should have the same detrimental impact on GPI‐anchored markers, GPI‐APs, as seen for pathogenic coding variants. So far, flow cytometry is used in a qualitative manner to assess GPI‐anchor deficiencies on a functional level. FACS analysis of the peripheral granulocytes is very helpful in the diagnostic procedure of inherited GPI deficiencies. Many IGD's show decreased expression of CD16 or FLAER compared with healthy controls. But the limitation is that the blood should be analyzed within 2 days without fixation, otherwise the expression levels gradually decrease, which makes the results unreliable. For more quantitatively applicable results, we established a flow cytometry protocol for fibroblasts and assessed the cell surface levels for GPI‐APs for different patients. In all three patients with noncoding mutations in *PGAP3*, the mean fluorescent intensity (MFI) of the GPI‐linked proteins CD55, CD59, as well as FLAER was markedly reduced compared with healthy controls (Fig. 3). The reduction was similar in PGAP3 deficient fibroblasts with compound heterozygous mutations (QM‐100:[p.Asp305Gly; p.Leu147Profs*16]) [Howard et al., 2014].

## Discussion

HPMRS represents the largest subgroup of IGDs. The clinical variability of the expressivity of this syndrome is wide and little is known about gene‐ or genotype–phenotype correlations. In our current study group intellectual disability as well as elevated ALP are the only consistent features across patients compared with other individuals affected with HPMRS caused by mutations in *PIGV, PIGW, PIGY*, *PIGO*, and *PGAP2*. Speech development, especially expressive language, was more affected than motor skills in the majority of patients (Table 1). Seizures are present in the majority of this cohort. Behavioral problems, in particular, sleep disturbances and autistic features as well as ataxia tend to be frequent. Most patients of this cohort presented with some facial dysmorphism compatible with HPMRS. Furthermore, brachytelephalangy, which is an important diagnostic sign in other types of HPMRS, is not present in any of the affected individuals with *PGAP3* mutations. Cleft palate as the only associated malformation found in this cohort has been previously documented in other individuals with HPMRS due to mutations in *PGAP3* but also in the other groups of Mabry syndrome. Variability regarding the postnatal growth, body weight, and OFC has already been observed in individuals carrying *PIGV*, *PIGO*, and *PGAP2* mutations.

For all known HPMRS disease genes, the majority of pathogenic variants are missense mutations, a finding that is also supported by our data. Frameshift mutations, which result most likely in a complete loss of function, have only been observed in a compound heterozygous state with missense mutations and individuals with such combinations seem to be affected more severely.

Flow cytometry of GPI‐anchored markers on the cell surface of peripheral granulocytes has been used in many patients with suspected IGDs for confirmatory diagnostic purposes. However, FACS measurements on blood cells are known to be variable in time and expression levels are difficult to compare between samples. In a measurement of granulocytes from F‐II‐3, we did, for instance, not observe a marked reduction of GPI‐makers, although the mutations have been shown to be pathogenic in the CHO test assay (Supporting Information). In other cases, repeated measurements of ALP in the same patient have also shown values that were only borderline high in some instances. Thus, also unremarkable results in flow cytometry of blood cells do not rule out HPMRS (Supp. Fig. S9).

For a better reproducibility and comparability of different pathogenic mutations, we established a FACS protocol for fibroblasts and tested cells with noncoding as well as coding mutations in *PGAP3* and *PIGV*. Although the numbers for which we were able to perform FACS analysis on fibroblasts are still too small to be statistically significant, there seems to be no obvious correlation between the MFIs of the tested markers and the severity of the phenotype. The reduction in MFI in fibroblasts with impaired PGAP3 is also comparable to cells with pathogenic mutations in *PIGV* that we previously assessed (PIGV‐X‐II‐1: [p.Ala341Glu; p.Ala341Glu], PIGV‐Y‐II‐1:[p.Ala165Glu; p.Arg460*], PIGV‐Z‐II‐2: [p.Cys156Tyr; p.Ala341Glu]). In two of these patients, intellectual disability is the predominant clinical feature [Krawitz et al., 2010; Horn et al., 2011], whereas in patient PIGV‐Y‐II‐1 (not reported so far) the most prominent manifestation is treatment‐resistant epilepsy. Seizures are a common feature in HPMRS and in some cases they are intractable and life‐threatening [Nakamura et al., 2014]; PIGV‐Y‐II‐1 thus represents a severely affected case with frequent status epilepticus. Still, no quantitative differences of the MFIs between the milder and more severe PIGV cases could be observed in the fibroblast cultures (data not shown).

Most pathogenic mutations in genes of the GPI‐anchor synthesis primarily decrease the surface expression of GPI‐anchored proteins (e.g., *PIGV*), whereas deficiencies in *PGAP2 or PGAP3* genes, which involve in lipid remodeling in the Golgi result in expression of GPI‐APs that are more prone to cleavage [Maeda et al., 2007]. *PGAP3* is among putative membrane‐bound hydrolases called CREST superfamily proteins, and it removes unsaturated fatty acid from the sn‐2 position of lipid moiety of GPI anchored protein in the Golgi, the first step of fatty acid remodeling, followed by the attachment of saturated fatty acid through *PGAP2* involvement [Pei et al., 2011]. This fatty acid remodeling reaction is necessary for association of GPI‐APs to lipid raft. In PGAP3‐deficient cells, GPI‐APs are expressed at normal level but could not localize on lipid rafts because of their non‐remodeled lipid structure, which might cause the symptoms of the patients.

For a better understanding of genotype–phenotype correlations in Mabry syndrome, it might be worthwhile to extend the flow cytometric profiling to more GPI‐APs and to further tissues. A deeper insight into the dynamics of GPI‐APs on the cell surface might also help us to design experiments for analyzing the conjectured feedback mechanism that regulates the expression of genes of the GPI‐anchor synthesis.

Our study also shows that new screening approaches, such as cDNA sequencing, will help increase the diagnostic yield for inherited GPI‐anchor deficiencies further. With a quantitative and allele‐specific analysis of transcripts, regulatory variants can be identified, extending the pathogenic mutation spectrum of Mabry syndrome.

## Supporting information

Disclaimer: Supplementary materials have been peer‐reviewed but not copyedited.

Figure S1: A‐II‐1
Figure S2: C‐II‐1
Figure S3: C‐II‐2
Figure S4: D‐II‐1
Figure S5: E‐II‐2 and E‐II‐4
Figure S6: PGAP3/2 double deficient CHO cells (C84 DM‐C2, Mol. Biol. Cell vol.18 1497‐1506,2007) were transiently transfected with HA‐tagged wild type and suggested mutant PGAP3 driven by the strong promoter SRalpha (pME HA‐PGAP3). Two days later, the surface expressions of GPI‐anchored proteins, CD59, DAF and uPAR were assessed by flow cytometry. PGAP2/3 double deficient cells which showed normal expression of GPI‐APs (dark gray shadows) became PGAP2 single deficient cells when it was transfected with wild type PGAP3 expression vector and surface expression of GPI‐APs were decreased (dotted lines). The activity of each mutant was decreased (thick lines).
Figure S7: Protein expression levels of PGAP3 wild type and mutant transcripts. Two days after transfection of each PGAP3 construct, lysates were immunoprecipitated with anti‐HA beads (H‐7, Sigma). The protein expression levels all mutants with the novel missense mutations C171R, L281P, D282G, S107L and W287C were significantly reduced. These results are comparable to mutant constructs with pathogenic mutations that have already been reported in (Howard, et al., 2014). The precipitates were digested with Endoglycosidase H or PNGase F. Only mature PGAP3 is resistant to Endo H. Thus a reduction of the smear of N‐glycosylated PGAP3 after Endo H indicates also a higher fraction of immature PGAP3 that resides in the ER.
Figure S8: PGAP3 expression measured by RT qPCR from fibroblast total RNA. Patient and control fibroblasts were cultured in 6‐wells for four days to reach full confluency. Total RNA was isolated with Trizol and 200ng of total RNA were used for reverse transcription to cDNA. GAPDH was used for normalization. Two biological replicates of RNA were used and qPCR were performed with triplicates. The expression of PGAP3 in the father of family B (B‐I‐1) is reduced by half, probably due to nonsense‐mediated decay of the transcript resulting from the c.402dupC mutation. The index (B‐II‐2) with the second mutation c.588‐10G>A shows a reduction of PGAP expression to only 10%. In family C the father (C‐I‐1) with the missense variant c.861G>T expresses PGAP3 at a similar level as healthy controls, while the mother (C‐I‐2) and the index (C‐II‐2) with the 3’ UTR mutation have substantially reduced expression of PGAP3. QM‐100 from Howard et al. (individual II‐1 from family B) shows also a reduction of PGAP3 expression by half, compared to pooled healthy controls.
Figure S9: Flow cytometric measurements of GPI‐linked markers on cell surface of blood cells. No marked reduction of expression levels compared to wild type controls. Black line: affected individual, dotted line: wild type control, gray area: unstained cells, background.
