# Supplementary material for: Rare Noncoding Mutations Extend the Mutational Spectrum in the PGAP3 Subtype of Hyperphosphatasia with Mental Retardation Syndrome
Source: Hum Mutat. 2016 May 19;37(8):737–44. doi: 10.1002/humu.23006 (PMC5084765; doi:10.1002/humu.23006)
Supplement: Supplementary file 1 — Figure S1: A‐II‐1 Figure S2: C‐II‐1 Figure S3: C‐II‐2 Figure S4: D‐II‐1 Figure S5: E‐II‐2 and E‐II‐4 Figure S6: PGAP3/2 double deficient CHO cells (C84 DM‐C2, Mol. Biol. Cell vol.18 1497‐1506,2007) were transiently transfected with HA‐tagged wild type and suggested mutant PGAP3 driven by the strong promoter SRalpha (pME HA‐PGAP3). Two days later, the surface expressions of GPI‐anchored proteins, CD59, DAF and uPAR were assessed by flow cytometry. PGAP2/3 double deficient cells which showed normal expression of GPI‐APs (dark gray shadows) became PGAP2 single deficient cells when it was transfected with wild type PGAP3 expression vector and surface expression of GPI‐APs were decreased (dotted lines). The activity of each mutant was decreased (thick lines). Figure S7: Protein expression levels of PGAP3 wild type and mutant transcripts. Two days after transfection of each PGAP3 construct, lysates were immunoprecipitated with anti‐HA beads (H‐7, Sigma). The protein expression levels all mutants with the novel missense mutations C171R, L281P, D282G, S107L and W287C were significantly reduced. These results are comparable to mutant constructs with pathogenic mutations that have already been reported in (Howard, et al., 2014). The precipitates were digested with Endoglycosidase H or PNGase F. Only mature PGAP3 is resistant to Endo H. Thus a reduction of the smear of N‐glycosylated PGAP3 after Endo H indicates also a higher fraction of immature PGAP3 that resides in the ER. Figure S8: PGAP3 expression measured by RT qPCR from fibroblast total RNA. Patient and control fibroblasts were cultured in 6‐wells for four days to reach full confluency. Total RNA was isolated with Trizol and 200ng of total RNA were used for reverse transcription to cDNA. GAPDH was used for normalization. Two biological replicates of RNA were used and qPCR were performed with triplicates. The expression of PGAP3 in the father of family B (B‐I‐1) is reduced by half, probably due to nonsense‐mediated deca [file HUMU-37-737-s001.pdf]

Supporting Information

Clinical Data

Patient A-II-1

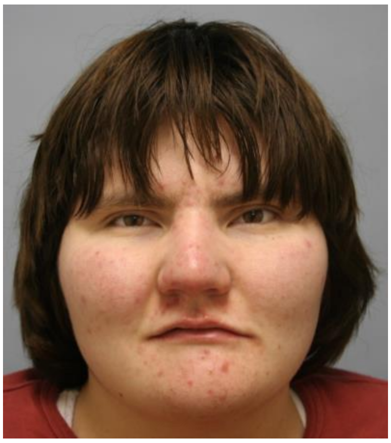

Supp. Figure S1: A-II-1

This patient is an adult female, the first child of non-consanguineous parents of European American origin. The further family history was unremarkable. She was delivered spontaneously at 40 weeks of gestation with a length of 49.5 cm (-1 SD), weight of 3175 g (-0.7 SD) and occipitofrontal head circumference (OFC) of 35 cm (mean). After birth, physical examination revealed a cleft palate, which had been surgically corrected in the first months of life. At that time, abdominal ultrasound and ultrasound examinations of the brain were normal. Echocardiogram revealed a small PDA that closed spontaneously.

Early developmental milestones were reported as normal with sitting at 6 months, walking at 13 months and first words at 9 months. At 2 years she has had regression in her vocabulary skills. Later on she had no verbal communication. At 3 years she developed attention deficit disorder with hyperactivity and significant sleeping problems. At 19 years, she was noted to have obsessive-compulsive disorder in addition to autistic features and constant sleep disturbance. At 20 years she has begun having seizures, which corresponded well to anticonvulsants.

During infancy and childhood, weight and length progressed in the normal range but head circumference was above the normal range. At age of 25 years, her length was 161.3 cm (- 0.6 SD), weight 87 kg, BMI 33.3 (obese range) and her OFC 58.2 cm (+3.2 SD). Her facial signs included synophris, deep set eyes, up-slanting palpebral fissures, a broad nasal bridge, high and prominent cheekbones, a short philtrum, thin upper and lower lips as well as bifid uvula (Supp. Figure S1). Her hands and feet were normal as well as her nails. X-ray of the hands revealed normal findings.

Apart from a variety of normal metabolic tests, AP activity was raised (469 U/L, upper limit of the normal range 136 U/L) in repeated tests.

Patient B-II-2

This girl is the second child of German parents. This pregnancy was complicated by gestational diabetes, and prolonged delivery necessitated caesarean delivery at week 38. Birth weight was 3950 g (+1.1 SD), length 49 cm (-0.5 SD) and normal head circumference.

Her early psychomotor development was normal. At age 16 months, she started walking. At age 12 months, the girl spoke her first word. Later on psychomotor retardation became evident with pronounced speech delay. Since the age of 18 months, myoclonic seizures occurred. At that age she developed significant sleep disturbance.

Physical examination of the 3-year-old patient showed a height of 97 cm (+0.4 SD), a weight of 18 kg (+ 2.4 SD), and an OFC of 50 cm (+0.4 SD) as well as muscular hypotonia and ataxia. Facial dysmorphism was subtle with up-slanting palpebral fissures, a short nose, broad nasal bridge and tip, tented upper lip and small teeth. Hands and feet showed broad finger and toe nails.

Apart from a small capsula interna the MRI investigation of the brain at age of 2 years gave a normal result. Serum alkaline phosphatase level was elevated with 333 U/L, upper normal range is 297 U/L at this age.

Patient C-II-1 and C-II-2

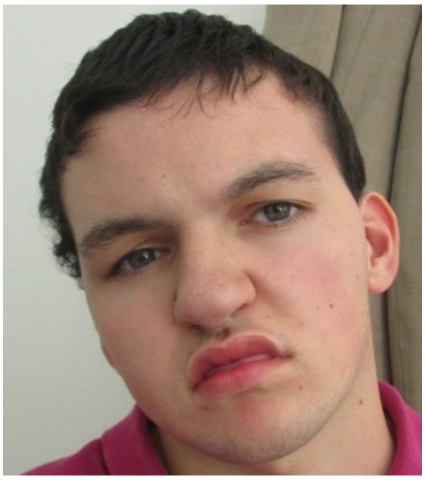

Supp. Figure S2: C-II-1

Patients C-II-1 and C-II-2 were first described by (Thompson, et al., 2010). The boy was born to unrelated healthy French parents after a term pregnancy and normal delivery (birth weight: 3370 g, height: 50 cm, OFC: 37 cm; +1.7 SD). His ability to sit unaided (12 months) and walk was delayed (after 3 years). After two years of age he received valproate and diazepam for iterative episodes of myoclonic jerks. At 5 years, examination showed normal height and weight but enlarged OFC (56.5 cm, +3 SD), coarse facies, long palpebral fissures, prominent cheeks, a large mouth with a tented upper lip, a short philtrum, and mild joint hypermobility (Supp. Figure S2). His gait was unstable with mild cerebellar ataxia. He had weak deep tendon reflexes. Oculomotor apraxia was noted. EEG and brain MRI were normal. Metabolic work up detected very high levels of total alkaline phosphatases (830-1000 U/l, normal range 125-410). Array CGH and metabolic tests were otherwise normal. He is now a 22 years-old man living in a home for disabled adults. He has limited autonomy, no speech development but he understands simple orders.

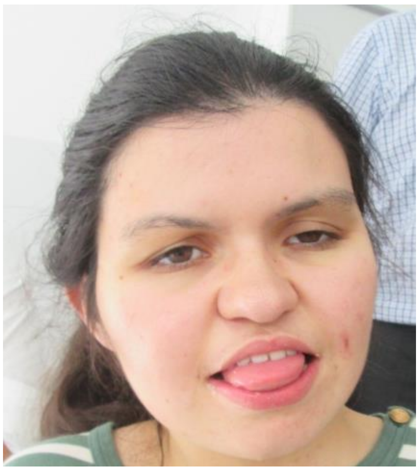

Supp. Figure S3: C-II-2

His younger sister was born after a term pregnancy and normal delivery (birth weight: 3300 g, height: 49 cm, OFC: 36.5 cm, +1.2SD). Her ability to sit unaided and walk was also delayed (3.5 years). At that age, her facial appearance was quite similar to that of her brother, with enlarged head circumference (53 cm, +2 SD), normal height and weight, long palpebral fissures, small ears and teeth, large mouth, full cheeks and mild joint hypermobility (Supp. Figure S3). She also had oculomotor apraxia and no speech development. Her gait was unstable with mild cerebellar ataxia. Her total plasma alkaline phosphatases were markedly elevated (830-900 U/l, normal range 125-410). Histopathological analyses of cultured skin fibroblasts and bone biopsies taken during hip surgery at 10 years revealed accumulation of mixed lipid and carbohydrate storage material of unknown nature in enlarged vacuoles within swollen cultured cells. At the age of 22 years, she had limited autonomy and no speech development.

Patient D-II-1

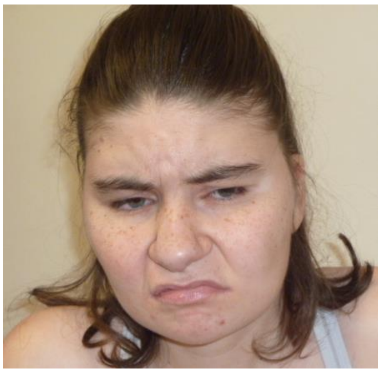

Supp. Figure S4: D-II-1

Her mother had four miscarriages but also a healthy son. In the pregnancy with the patient the mother described reduced fetal movements. The patient was delivered by planned Caesarean section at 40 weeks, weighing 4300 g (+2 SD). A Cleft soft palate was repaired at 7 months of age. She sat unstably at 9 months and walked at three years, but was extremely unsteady and slow. She has never spoken and is not continent. Abdominal pain and chronic constipation led to the diagnosis of intestinal malrotation, which was repaired at the age of 9 years, but constipation did not improve. She started her menses at the age of 12 years. During her teenage years she developed autistic features and episodes of hypersomnolence classified as Kleine-Levin syndrome. Therefore she was treated with carbamazepine for 10 years. After stopping of this therapy at age of 23 years, she developed seizures. Examination at this age showed normal OFC (55.6 cm, +1.2 SD), slightly coarse face with deep set eyes and prominent cheeks (Supp. Figure S4). Her hands and feet appeared normal, however her second toenail was broad. In repeated tests she has been found to have increased AP (667 U/L with normal range 35-105).

Patient E-II-2 and E-II-4

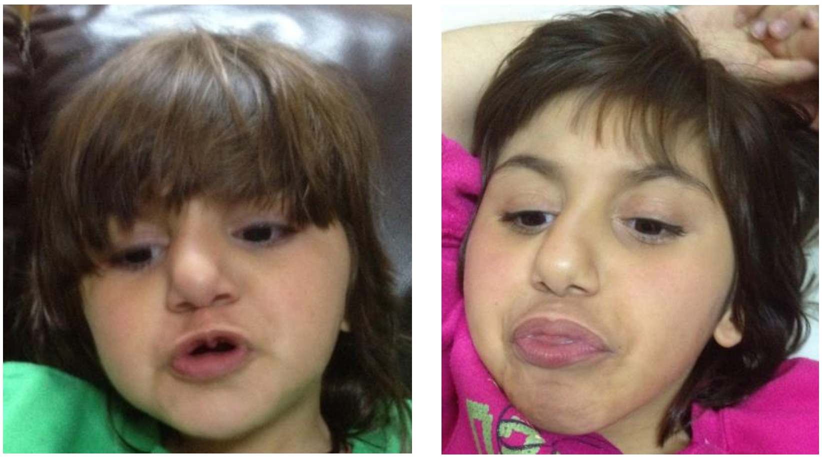

Supp. Figure S5: E-II-2 and E-II-4

These two girls are the second and forth child of consanguineous Palestinian parents. Both pregnancies were normal with spontaneous term delivery without complications, length, weight and OFC within normal limits. Both affected siblings had early developmental delay with severe lax ligaments and muscular hypotonia. Psychomotor retardation became evident with pronounced speech delay and later on they developed severe spastic quadriparesis. E-II-2 remained bed ridden and can use her hand only for simple tasks. E-II-4 started walking with help at age of 7 years. Brain MRIs of both patients at age of 2 and 3 years showed brain atrophy of temporal lobes partially due to vascular events. Since the age of 5 years tonic clonic seizures occurred in E-II-2 and had been very difficult to manage by different anti-epileptic drugs (Lamotrigene, clonazepam). At that E-II-2 also developed significant sleep disturbance treated by melatonin. EEG showed generalized slowing of the background with spike/slow wave activity and focal epileptic activity most highlighted in occipital area. Facial features included a broad nasal tip and a tented upper lip in both patients (Supp. Figure S5)

Patient F-II-3

This boy is the third child of non-consanguineous Japanese parents. His brother and sister are both healthy. He was born at 34 weeks gestation by caesarean section. Cleft palate without cleft lip, macroglossia, micrognathia, and ear lobe anomaly were noticed at birth. Due to respiratory distress and poor sucking, he needed intensive medical care, and required tube feeding until 1.5 months. He had severe muscular hypotonia and sensorineural hearing loss. There was no brachytelephalangy. His psychomotor development was severely delayed. He learned to roll over at 8 months. At last examination at age of 4 years and 4 months he could not sit alone. He was always very irritable and had no meaningful words. No seizure occurred. Sleep disturbance and bruxism were noted. He had recurrent episodes of pneumonia and suffered from severe food allergy and eczema.

Increased serum alkaline phosphatase was persistent (1,848 to 5,275 IU/L, compared to normal range 115 to 335 IU/L). High serum IgE was also noted (220 to 680 IU/mL, compared to normal range <170 IU/mL). Flow cytometry showed normal expression of GPI-APs on blood cells including erythrocytes, granulocytes, lymphocytes, and monocytes (Supp. Figure S6) Head MRI revealed megacisterna magna. Body CT scan and ultrasonography revealed no apparent morphological anomaly.

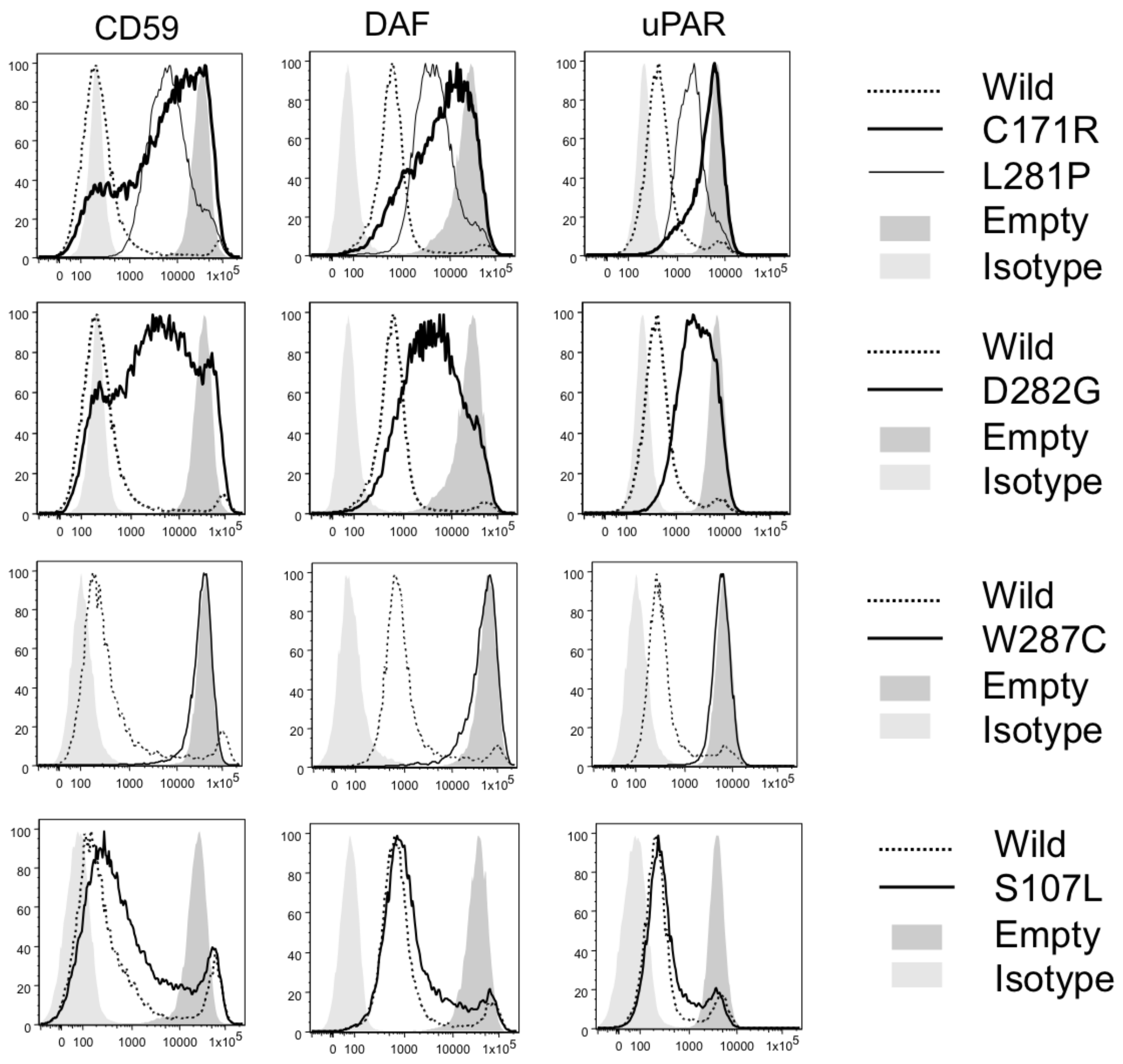

Supp. Figure S6: PGAP3/2 double deficient CHO cells (C84 DM-C2, *Mol. Biol. Cell* vol.18 1497-1506,2007) were transiently transfected with HA-tagged wild type and suggested mutant PGAP3 driven by the strong promoter SRalpha (pME HA-PGAP3). Two days later, the surface expressions of GPI-anchored proteins, CD59, DAF and uPAR were assessed by flow cytometry. PGAP2/3 double deficient cells which showed normal expression of GPI-APs (dark gray shadows) became PGAP2 single deficient cells when it was transfected with wild type PGAP3 expression vector and surface expression of GPI-APs were decreased (dotted lines). The activity of each mutant was decreased (thick lines).

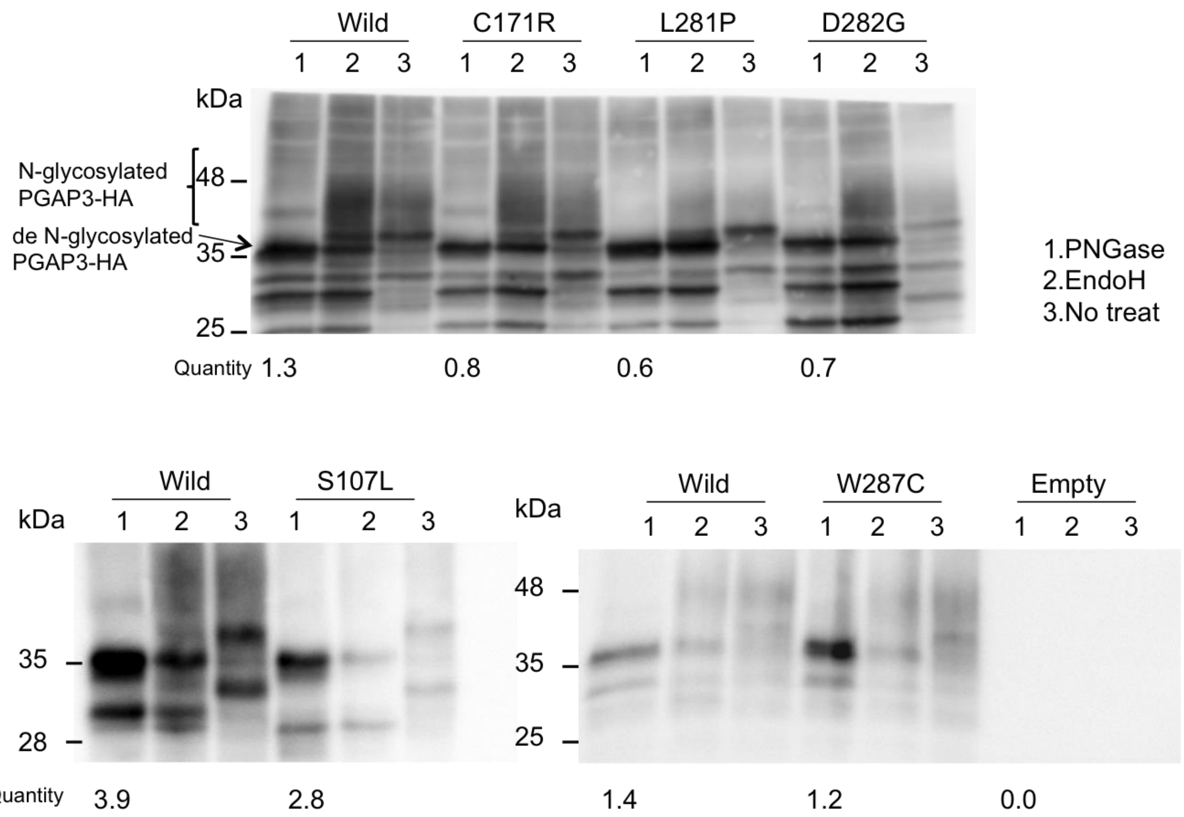

Supp. Figure S7: Protein expression levels of PGAP3 wild type and mutant transcripts. Two days after transfection of each PGAP3 construct, lysates were immunoprecipitated with anti-HA beads (H-7, Sigma). The protein expression levels all mutants with the novel missense mutations C171R, L281P, D282G, S107L and W287C were significantly reduced. These results are comparable to mutant constructs with pathogenic mutations that have already been reported in (Howard, et al., 2014). The precipitates were digested with Endoglycosidase H or PNGase F. Only mature PGAP3 is resistant to Endo H. Thus a reduction of the smear of N-glycosylated PGAP3 after Endo H indicates also a higher fraction of immature PGAP3 that resides in the ER.

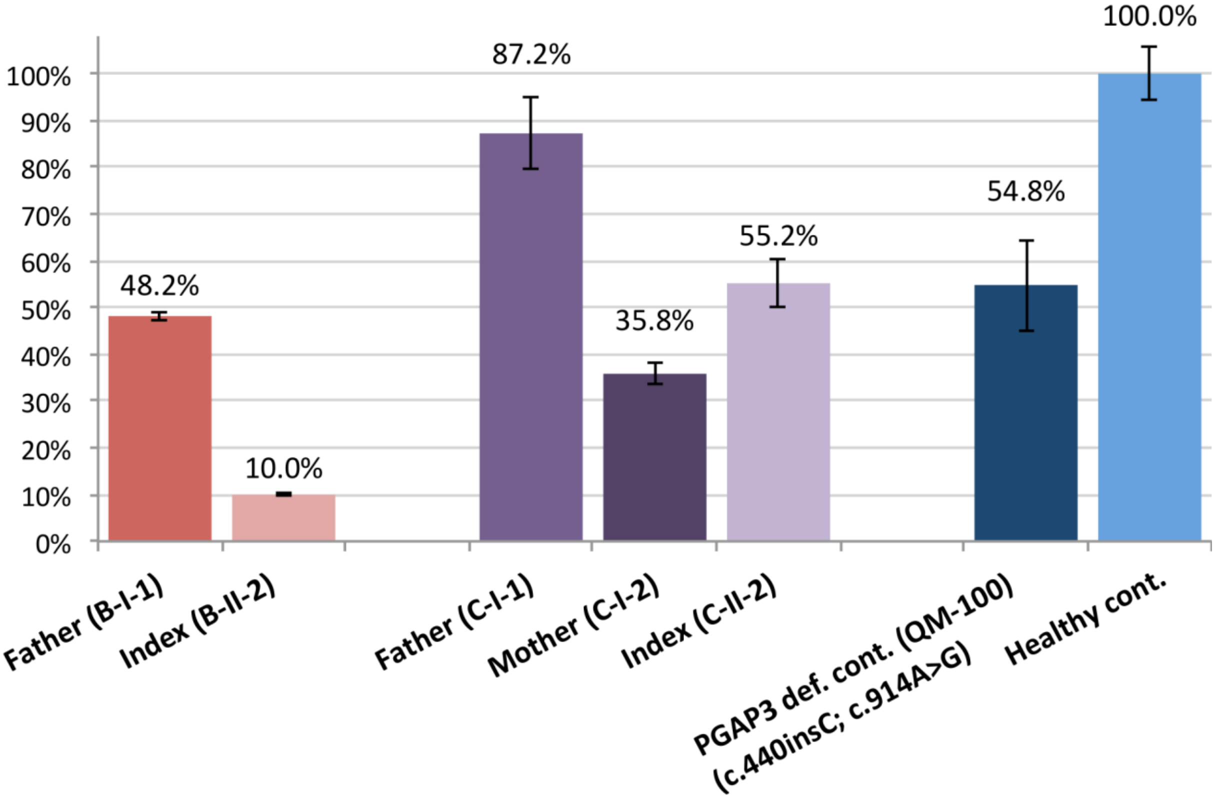

Supp. Figure S8: *PGAP3* expression measured by RT qPCR from fibroblast total RNA. Patient and control fibroblasts were cultured in 6-wells for four days to reach full confluency. Total RNA was isolated with Trizol and 200ng of total RNA were used for reverse transcription to cDNA. GAPDH was used for normalization. Two biological replicates of RNA were used and qPCR were performed with triplicates. The expression of *PGAP3* in the father of family B (B-I-1) is reduced by half, probably due to nonsense-mediated decay of the transcript resulting from the c.402dupC mutation. The index (B-II-2) with the second mutation c.588-10G>A shows a reduction of *PGAP* expression to only 10%. In family C the father (C-I-1) with the missense variant c.861G>T expresses PGAP3 at a similar level as healthy controls, while the mother (C-I-2) and the index (C-II-2) with the 3' UTR mutation have substantially reduced expression of PGAP3. QM-100 from Howard et al. (individual II-1 from family B) shows also a reduction of PGAP3 expression by half, compared to pooled healthy controls.

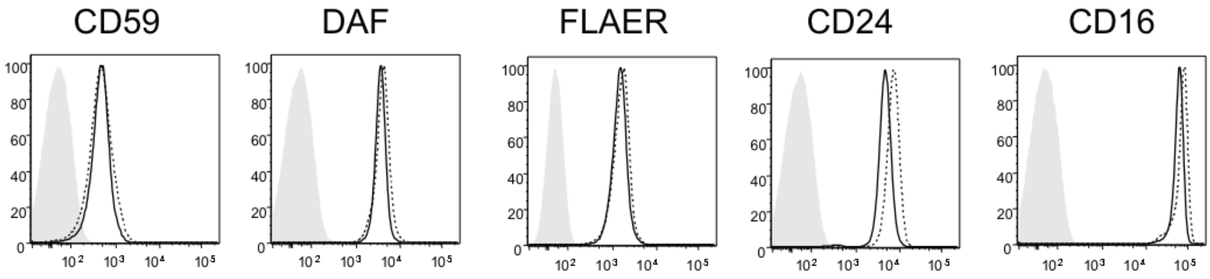

Supp. Figure S9: Flow cytometric measurements of GPI-linked markers on cell surface of blood cells. No marked reduction of expression levels compared to wild type controls. Black line: affected individual, dotted line: wild type control, gray area: unstained cells, background.

Supp. References

Howard MF, Murakami Y, Pagnamenta AT, Daumer-Haas C, Fischer B, Hecht J, Keays DA, Knight SJ, Kolsch U, Kruger U and others. 2014. Mutations in PGAP3 Impair GPI-Anchor Maturation, Causing a Subtype of Hyperphosphatasia with Mental Retardation. Am J Hum Genet.

Thompson MD, Nezarati MM, Gillessen-Kaesbach G, Meinecke P, Mendoza-Londono R, Mornet E, Brun-Heath I, Squarcioni CP, Legeai-Mallet L, Munnich A and others. 2010. Hyperphosphatasia with seizures, neurologic deficit, and characteristic facial features: Five new patients with Mabry syndrome. Am J Med Genet A 152A(7):1661-9.
